# Prevalence and Size-Based Risk Categorization of Pancreatic Cysts Among Asymptomatic Individuals With Screening MRI

**DOI:** 10.1001/jamanetworkopen.2026.0983

**Published:** 2026-03-09

**Authors:** Paul Wong, Tommaso Pollini, Sophia Hernandez, Marco Zampese, Letizia Todeschini, Luis Laurean Aguilar, Saqib Abdullah Basar, Sam Hashemi, Ajay V. Maker

**Affiliations:** 1 Division of Surgical Oncology, Department of Surgery, University of California, San Francisco, San Francisco; 2 Prenuvo Inc, Vancouver, British Columbia, Canada

## Abstract

**Importance:**

Subsets of pancreatic cystic lesions (PCLs) are the only radiographically evident precursors to pancreatic cancer. Guidelines prioritize cyst size and high-risk features to determine the need for resection. However, the true prevalence of PCLs and risk factors for their development in the general population remain unknown, as available data are often biased by imaging performed in symptomatic patients.

**Objective:**

To determine prevalence of PCLs in an asymptomatic North American cohort using size-based risk categorization and identify associated demographic factors and exposures.

**Design, Setting, Participants:**

This cross-sectional study included asymptomatic individuals who underwent rapid whole-body magnetic resonance imaging using a commercially available 1.5T device for general preventive screening between January 1, 2020, and May 31, 2023. Scans were interpreted by certified radiologists using standardized synoptic reporting templates. Diagnostic free-text reports were analyzed, and positive reports underwent independent additional review. Data were analyzed from February 4, 2024, to March 6, 2025.

**Exposures:**

Participant demographic information and lifestyle exposures.

**Main Outcomes and Measures:**

Prevalence of PCLs based on size-based risk categorization.

**Results:**

Among 21 651 individuals undergoing screening MRI (11 050 [51.0%] male; median age, 51 [IQR, 42-61] years), 1509 (7.0%) had an incidental PCL (776 [51.4%] female), and age- and sex-standardized prevalence was 6.3%. The median age was higher in individuals with PCLs (61 [IQR, 51-69] vs 50 [IQR, 41-60] years). Prevalence increased with age, from 86 of 4223 (2.0%) 39 years or younger to 229 of 5720 (4.0%) aged 40 to 49 years, 390 of 5586 (7.0%) aged 50 to 59 years, 467 of 4049 (11.5%) aged 60 to 65 years, 268 of 1741 (15.4%) aged 70 to 79 years, and 69 of 332 (20.8%) 80 years or older (*P* < .001). Most cysts (1449 [96.0%]) were less than 2 cm, 1200 (79.5%) were less than 1 cm, and 17 (1.1%) were 3 cm or larger (0.08% of all scans). Independent factors associated with PCLs included being 65 years or older (odds ratio [OR], 3.03; 95% CI, 2.67-3.45), female sex (OR, 1.13; 95% CI, 1.002-1.27), personal history of pancreatitis (OR, 2.65; 95% CI, 1.66-4.08) or pancreatic ductal adenocarcinoma (PDAC) (OR, 20.98; 95% CI 3.70-161.45), family history of PDAC (OR, 1.40; 95% CI, 1.04-1.86), alcohol consumption (OR, 1.15; 95% CI, 1.01-1.31), and Latin American (OR, 1.79; 95% CI, 1.43-2.23) or Middle Eastern (OR, 1.40; 95% CI, 1.03-1.86) ethnicity. Asian ethnicity (OR, 0.79; 95% CI, 0.66-0.95) was associated with lower PCL prevalence.

**Conclusions and Relevance:**

In this cross-sectional study of 21 651 individuals, incidental PCLs were common (6.3%) and found in as many as 20.8% of older individuals undergoing whole-body MRI screening, with 1492 PCLs (98.9%) of all PCLs being less than 3 cm.

## Key Points

**Question:**

What is the prevalence of clinically relevant pancreatic cystic lesions (PCLs) among asymptomatic adults who undergo screening with whole-body magnetic resonance imaging (MRI)?

**Findings:**

In this cross-sectional study of 21 651 North American individuals with whole-body MRI screening, the standardized prevalence of PCLs was 6% overall and 21% in individuals 80 years and older. Older age, female sex, history of pancreatitis and pancreatic ductal adenocarcinoma, family history of pancreatic ductal adenocarcinoma, alcohol use, and Latin American or Middle Eastern ethnicity were associated with higher likelihood of cyst development; 99% of lesions fell below the size threshold associated with a worrisome feature of increased malignant potential (3 cm).

**Meaning:**

These findings suggest that most incidental PCLs, while common, may be very low risk based on size-based risk categorization alone.

## Teaser

This cross-sectional study assesses the prevalence of pancreatic cystic lesions in a North American cohort of asymptomatic patients undergoing screening with whole-body magnetic resonance imaging, estimates risk using size-based categorization, and identifies associated demographic factors and exposures.

## Introduction

Pancreatic cystic lesions (PCLs) range from simple cysts to premalignant neoplasms, including intraductal papillary mucinous neoplasms (IPMNs) and mucinous cystic neoplasms. While most PCLs are benign, a small proportion may progress to malignancy.^1,2^ Due to advances in imaging technology and widespread use of cross-sectional imaging, PCLs are being identified more frequently, often incidentally. Incidentally found PCLs may lead to immense anxiety for patients and prompt a cascade of further medical investigation due to concerns regarding malignancy.^3^ As cross-sectional imaging, specifically magnetic resonance imaging (MRI), is being increasingly pursued for voluntary preventive health screening, the identification of PCLs may increase further, leading to increased anxiety and potentially overutilization of health care resources if appropriate management strategies are not implemented. The true prevalence and potential health care burden of PCL remains unknown in a healthy population.

Previous studies have used computed tomography (CT) scans to assess the prevalence of PCLs, but MRI may offer greater sensitivity.^4,5,6,7^ As a result, several societies recommend MRI over CT for PCL surveillance.^8,9^ Vilela et al^10^ conducted a meta-analysis on PCL prevalence using MRI; however, most of the studies were limited by their inclusion of symptomatic patients or individuals undergoing imaging for other conditions, which raises concerns about their applicability to the broader asymptomatic population. Similarly, Kimura et al^11^ have looked at the prevalence of PCLs through autopsy cases, but these reflect older, hospitalized populations rather than healthy adults living in the community. Collectively, these limitations make it difficult to generalize prior prevalence estimates to the general population. To our knowledge, no study has examined PCL prevalence in an asymptomatic North American population undergoing screening MRI. Understanding the population level prevalence of PCLs is critical to informing surveillance strategies, resource utilization, and risk stratification. Prior prevalence estimates have varied widely, reflecting heterogeneity in study populations, imaging modalities, age distributions, and ascertainment methods. As a result, existing studies provide limited insight into the true prevalence of pancreatic cysts in contemporary, asymptomatic populations undergoing screening imaging. More accurate prevalence estimates in well-characterized screening cohorts are essential to contextualize incidental findings, guide clinical decision making, and anticipate the downstream impact of evidence-based screening and surveillance recommendations.

Beyond prevalence, population-level analyses have not thoroughly investigated the demographic factors, lifestyle exposures, and familial predispositions associated with PCL development. Understanding these risk factors is increasingly important as the detection of PCLs continues to rise. Moreover, it is important to recognize the proportion of these PCLs that are clinically relevant based on International Association of Pancreatology (IAP)–defined worrisome features.^12,13,14^ This knowledge is essential for refining clinical management strategies and guiding decisions on whether to pursue further diagnostic evaluations when PCLs are incidentally discovered. Thus, this study aimed to determine the prevalence of PCLs in a North American cohort of asymptomatic individuals, estimate risk using only size-based categorization, and identify associated demographic factors and exposures using a large dataset of high-resolution screening MRIs.

## Methods

This cross-sectional study was not considered human participant research and therefore did not require review or informed consent per the Common Rule. This report followed the Strengthening the Reporting of Observational Studies in Epidemiology (STROBE) reporting guideline.

### Study Sample

Adults (age range, 18-97 years) who voluntarily underwent rapid whole-body imaging on a commercially available 1.5T MRI device for general preventative screening between January 1, 2020, and May 31, 2023, were included (Prenuvo Inc). Before undergoing imaging, individuals completed an extensive intake questionnaire that captured detailed demographic information, including sex, race and ethnicity (categorized as American Indian or Alaska Native, Asian, Black, Indigenous, Latin American, Middle Eastern, White, and unknown), medical history, and lifestyle factors. Race and ethnicity data were collected to analyze for potential association of PCLs with people of certain backgrounds. The definition of vigorous exercise was adopted from the Centers for Disease Control and Prevention as any activity that prevents individuals from “saying more than a few words without pausing for a breath.”^15^ Individuals were asked about any history of tobacco smoking and active alcohol use within the last year.

Scans were interpreted by board-certified radiologists with expertise in whole-body MRI. Each scan was assessed using a standardized synoptic reporting template designed to ensure consistency and comprehensiveness in documenting findings. Within this synoptic report, radiologists selected from a predefined list of diagnostic conditions that best represented the imaging finding. These conditions included cystic lesions, IPMNs, ductal adenocarcinoma, fatty infiltration, endocrine tumors, pancreatitis, pancreatic divisum, or other. Radiologists used the option of other if they preferred to dictate a free-text diagnostic impression or had a nuanced finding to describe that was not optimally captured by the templated condition options.

Cases that were labeled as other were subjected to a natural language search of relevant terms to ensure comprehensiveness of case extraction from the reports. Keywords used to identify PCLs described in the other category included *cystic lesion*, *indeterminate*, *IPMN*, *intraductal papillary mucinous neoplasm*, *side branch*, *main duct*, *mixed type*, *solid cystic lesion*, *complex cystic lesion*, *cyst*, *pseudocyst*, *serous cystadenoma*, *mucinous cystic neoplasm*, *thickened cyst wall*, *main duct dilatation*, *connecting side branch*, *segmental dilatation*, *main pancreatic duct*, *solid component*, *mural nodule*, and *septation*. All radiologic reports selected from this keyword search underwent subsequent manual review for accuracy. PCLs were chosen for this study if they were labeled on the synoptic report as cystic lesions, IPMNs, or those deemed appropriate from the cases labeled other. Size measurements of PCLs were recorded from the synoptic reports, and for any PCL that lacked a specific size measurement in the radiologist’s report, manual independent MRI re-review and cyst measurement were performed.

### Statistical Analysis

Data were analyzed from February 4, 2024, to March 6, 2025. Categorical variables were presented as proportions, and continuous variables were reported as median and IQR. Proportions between groups were compared using the χ^2^ test. Medians of continuous variables were compared using the Mann-Whitney test. Multivariable associations were evaluated using lasso-penalized logistic regression to reduce model overfitting and perform variable selection in the setting of multiple candidate factors associated with PCLs. The model included the following prespecified variables based on clinical relevance: 65 years or older, sex, body mass index (calculated as weight in kilograms divided by height in meters squared) greater than 30, history of pancreatitis, personal history of pancreatic ductal adenocarcinoma (PDAC) or any non-PDAC malignant neoplasm, family history of PDAC, ethnicity, smoking history, alcohol use, and vigorous exercise. The optimal penalty parameter (λ) was selected using cross-validation. This approach was chosen to avoid overinterpreting statistically significant but clinically trivial differences in the context of our large sample size and to focus inference on variables with meaningful effect sizes. Adjusted odds ratios (ORs) and 95% CIs were reported.

To account for differences in the age and sex distribution between the study cohort and the general adult population, the age- and sex-standardized prevalence of PCLs was calculated. Participants were grouped into 6 age categories (≤39, 40-49, 50-59, 60-69, 70-79, and ≥80 years) and by sex. Within each age and sex stratum, the observed prevalence was calculated as the proportion of participants with PCLs. Stratum-specific prevalence estimates were then weighted by the corresponding proportion of the US adult population in each age and sex group, based on US Census data,^16^ and summed to generate an overall standardized prevalence. Strata with missing data were excluded, and weights were renormalized to sum to 1.

Variables were included in the model if they were clinically relevant and statistically significant on univariable analysis. Statistical significance was set at a 2-sided *P* < .05. All statistical analyses were performed using R software, version 4.4.2 (R Project for Statistical Computing).

## Results

**Figure 1.**
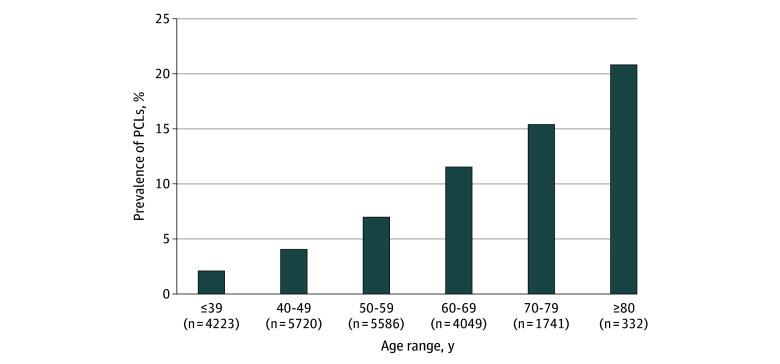
Bar Graph Showing Prevalence of Pancreatic Cystic Lesions (PCLs) Stratified by Age

Of the 21 651 individuals who underwent whole-body MRI (10 601 [49.0%] female and 11 050 [51.0%] male; median age, 51 [IQR, 42-61] years), 1509 (7.0%) were found to have a PCL. Age and sex standardization using population weights was conducted, and the standardized prevalence of PCLs was 6.3%. In terms of race and ethnicity, 184 participants (0.8%) were American Indian or Alaska Native; 3306 (15.3%), Asian; 189 (0.9%), Black; 936 (4.3%), Latin American; 672 (3.1%), Middle Eastern; 14 541 (67.2%), White; and 1823 (8.4%), unknown. The median age of individuals with PCLs was significantly higher (61 [IQR, 51-69] years) compared with those without PCLs (50 [IQR, 41-60] years; *P* < .001). The prevalence of PCLs was 567 of 3805 (14.9%) in individuals 65 years or older compared with 942 of 17 846 (5.3%) in those younger than 65 years. Additionally, PCL prevalence increased progressively with age, from 86 of 4223 (2.0%) 39 years or younger, 229 of 5720 (4.0%) aged 40 to 49 years, 390 of 5586 (7.0%) aged 50 to 59 years, 467 of 4049 (11.5%) aged 60 to 69 years, 268 of 1741 (15.4%) aged 70 to 79 years, and 69 of 332 (20.8%) 80 years or older (Figure 1).

On univariable analysis, individuals with PCLs were more likely to be female (776 [51.4%] vs 9825 [48.8%]) and to have a history of pancreatitis (28 [1.9%] vs 148 [0.7%]), personal history of PDAC (5 [0.3%] vs 2 [0.01%]) or non-PDAC cancers (253 [16.8%] vs 2211 [11.0%]), family history of PDAC (59 [3.9%] vs 599 [3.0%]), and history of alcohol consumption (1044 [69.2%] vs 13 150 [65.3%]) (Table). Conversely, those with PCLs were less likely to engage in vigorous exercise (671 [44.5%] vs 9773 [48.5%]). No associations were found between BMI or smoking history and PCL prevalence.

**Table.** Patient Demographics and Exposures Associated With the Development of PCLs

Characteristic	Univariable analysis, No. (%)			Multivariable analysis	
	PCL (n = 1509)	No PCL (n = 20 142)	*P* value	OR (95% CI)	*P* value
Aged ≥65 y	567 (37.6)	3238 (16.1)	<.001	3.03 (2.67-3.45)	<.001
Sex					
Male	733 (48.6)	10 317 (51.2)	.047	1 [Reference]	NA
Female	776 (51.4)	9825 (48.8)		1.13 (1.002-1.27)	.046
BMI ≥30	211 (14.0)	2711 (13.5)	.75	0.97 (0.83-1.14)	.73
History of pancreatitis	28 (1.9)	148 (0.7)	<.001	2.65 (1.66-4.08)	<.001
Personal history of PDAC	5 (0.3)	2 (0.01)	<.001	20.98 (3.70-161.45)	<.001
Personal history of any non-PDAC cancer	253 (16.8)	2211 (11.0)	<.001	1.13 (0.96-1.32)	.15
Family history of PDAC	59 (3.9)	599 (3.0)	.04	1.40 (1.04-1.86)	.02
Race and ethnicity					
American Indian or Alaska Native	12 (0.8)	172 (0.9)	<.001	0.77 (0.36-1.45)	.46
Asian	171 (11.3)	3135 (15.6)		0.79 (0.66-0.95)	.01
Black	13 (0.9)	176 (0.9)		0.80 (0.40-1.42)	.48
Latin American	114 (7.6)	822 (4.1)		1.79 (1.43-2.23)	<.001
Middle Eastern	62 (4.1)	610 (3.0)		1.40 (1.03-1.86)	.02
White	1032 (68.4)	13 509 (67.1)		1 [Reference]	NA
Unknown	105 (7.0)	1718 (8.5)			
History of any smoking	449 (29.8)	5714 (28.4)	.38	0.93 (0.82-1.06)	.28
Active alcohol consumption within last year	1044 (69.2)	13 150 (65.3)	.04	1.15 (1.01-1.31)	.03
Vigorous exercise	671 (44.5)	9773 (48.5)	<.001	0.98 (0.86-1.10)	.71

Abbreviations: BMI, body mass index (calculated as weight in kilograms divided by height in meters squared); NA, not applicable; PCL, pancreatic cystic lesion; PDAC, pancreatic ductal adenocarcinoma.

**Figure 2.**
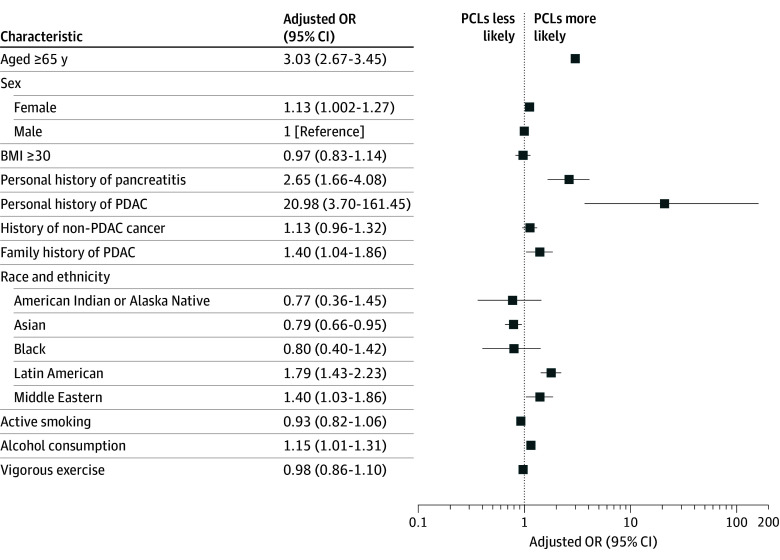
Forest Plot Depicting Factors Associated With Pancreatic Cystic Lesions (PCLs)

Multivariate analyses identified several independent factors associated with harboring a PCL (Table and Figure 2). Being 65 years or older (OR, 3.03; 95% CI, 2.67-3.45; *P* < .001), female sex (OR, 1.13; 95% CI, 1.002-1.27; *P* = .046), personal history of pancreatitis (OR, 2.65; 95% CI, 1.66-4.08; *P* < .001), personal history of PDAC (OR, 20.98; 95% CI, 3.70-161.45; *P* < .001), family history of PDAC (OR, 1.40; 95% CI, 1.04-1.86; *P* = .02), alcohol consumption (OR, 1.15; 95% CI, 1.01-1.31; *P* = .03), and Latin American (OR, 1.79; 95% CI, 1.43-2.23; *P* < .001) or Middle Eastern (OR, 1.40; 95% CI, 1.03-1.86; *P* = .02) ethnicity were independently associated with increased PCL prevalence. In contrast, Asian ethnicity (OR, 0.79; 95% CI, 0.66-0.95; *P* = .01) was associated with a decreased prevalence of PCLs. BMI, smoking history, history of non-PDAC cancers, and vigorous exercise did not reveal associations on multivariate analysis.

**Figure 3.**
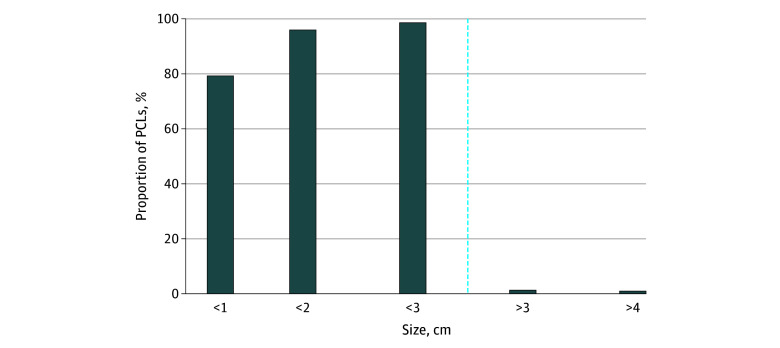
Bar Graph Showing Proportion of Individuals With Measured Pancreatic Cystic Lesion (PCL) Sizes The dashed vertical line indicates PCLs of 3 cm; 3 cm or greater is defined as a worrisome feature by the International Association of Pancreatology.

**Figure 4.**
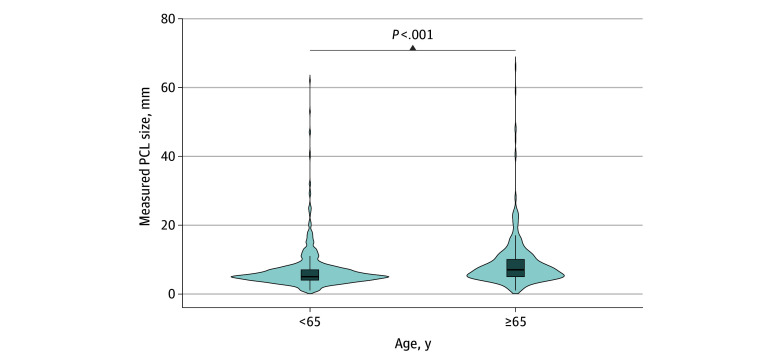
Violin Plot Demonstrating the Distribution of Measured Pancreatic Cystic Lesion (PCL) Sizes by Age Horizontal center lines indicate medians; boxes, IQRs. Whiskers extend to the most extreme values within 1.5 × IQR. Values beyond this range are plotted individually as outliers.

Regarding cyst sizes, 1200 (79.5%) were less than 1 cm, 1449 (96.0%) were less than 2 cm, and 1492 (98.9%) were less than 3 cm (Figure 3). Among all scans (n = 21 651), 17 (0.08%) and 14 (0.06%) revealed a lesion greater than 3 cm and greater than 4 cm, respectively. The distribution of cyst sizes differed significantly between individuals by age (Figure 4), with a median cyst size of 5 (IQR, 4-7) mm in those younger than 65 years and 7 (IQR, 5-10) mm in those 65 years or older (*P* < .001).

## Discussion

To our knowledge, this is the largest study to assess the prevalence of PCLs in individuals using screening MRI and the only study of its kind in a North American cohort. Among 21 651 individuals undergoing whole-body MRI for preventative screening, PCLs were identified incidentally in 7.0%, with an age- and sex-standardized prevalence of 6.3%. Prevalence increased progressively with age, from 2.0% in individuals 39 years or younger to 20.8% in those 80 years or older. These findings align with those of existing literature, which similarly demonstrates an age-related increase.^17^

The prevalence of PCLs observed in the present study falls within the broad range reported in the literature but differs from prior estimates depending on study population and imaging modality.^4,17,18^ MRI-based studies in Western populations have reported prevalence rates ranging from approximately 2% to 49%, with higher estimates generally observed in older cohorts and when MRCP sequences are routinely included or scans are reviewed again. In contrast, CT-based cohorts have reported lower prevalence estimates, likely reflecting lower sensitivity of CT for small or septated cystic lesions.^4^ Differences between the prevalence observed in this study and those reported in MRI-based Asian cohorts may be attributable to several factors, including ethnic and geographic variability, differences in age distribution, indications for imaging, and imaging protocols. As demonstrated in the present analysis, prevalence varies substantially by ethnicity, which may partially account for different prevalence reported in some cohorts. Additionally, prior studies have variably included symptomatic patients, individuals undergoing imaging for other clinical indications, or autopsy cohorts, each of which may inflate prevalence estimates relative to a more screening-based cohort or younger population. Importantly, to our knowledge, only 4 prior studies^19,20,21,22^ have evaluated screening MRI in largely healthy, asymptomatic populations undergoing whole body or abdominal MRI as part of comprehensive screening program. These studies reported prevalence estimates of approximately 2.4% in a Dutch cohort (n = 2803),^19^ 1% in a Turkish cohort (n = 103),^20^ 13.7% in a Japanese cohort (n = 5296),^21^ and 49% in a German cohort (n = 1077),^22^ highlighting the marked heterogeneity across populations and screening protocols. Differences between these cohorts and the present study may reflect ethnic and geographic variability, age distribution, imaging protocols, and ascertainment methods, all of which are known to influence prevalence estimates.

Patient demographic, medical, and lifestyle factors were analyzed to identify factors associated with PCLs. Advanced age, female sex, personal history of pancreatitis or PDAC, familial history of PDAC, and alcohol consumption were associated with an increased likelihood of PCLs. While age, history of pancreatitis, and alcohol consumption are established risk factors for PCL development,^4,5,23^ limited data exist regarding the association between PCLs and prior non-PDAC malignant neoplasms, which was not observed in this study; however, the prevalence of PCLs was significantly increased among participants with a history of non-PDAC cancer in the univariate analysis. Given that PCLs are recognized precursors to PDAC,^24,25^ individuals with a history of non-PDAC cancer may have underlying genetic predispositions that increase their risk of developing PCLs, though this association remains poorly understood. Although certain pancreatic cyst subtypes are more commonly observed in females, prior population-based studies have not demonstrated a consistent sex-specific difference in the overall prevalence of PCLs^10,26^; however, in the present study population, PCLs were modestly more prevalent among females.

Additionally, the observed association between PCLs and a family history of PDAC is consistent with findings from the Cancer of the Pancreas Screening (CAPS) study group.^27,28,29^ However, whether a family history of PDAC independently increases the risk of pancreatic cancer in patients with PCLs remains uncertain, as prior studies have reported conflicting results.^30,31^ Last, this study identified an increased likelihood of PCLs in individuals of Latin American and Middle Eastern descent, whereas Asian ethnicity was associated with decreased odds of PCL development.

In this study, 98.9% of identified PCLs were smaller than 3 cm. This size threshold is clinically important, as the IAP guidelines classify cysts 3 cm or larger as a worrisome feature for IPMNs, influencing individualized surveillance strategies.^14^ For cysts smaller than 2 cm—which accounted for 96.0% of PCLs in this study—the Kyoto guidelines^14^ propose eventual surveillance cessation if the lesion remains morphologically stable and lacks worrisome features. This evolving approach is supported by evidence revealing that the malignant progression risk for branch-duct IPMNs without worrisome or high-risk features is comparable to the general population, particularly in patients older than 75 years with cysts smaller than 3 cm and those older than 65 years with cysts 1.5 cm or smaller following 5 years of stability.^32,33^ Determining when to stop scanning patients is increasingly urgent, with real implications for patient quality of life and use of health care resources. Ultimately, for stable and small cysts, consideration of geography, sex, and individual physiologic reserve should guide cessation of imaging.^33^

Most of the cysts identified in this study fall below the size threshold associated with increased malignant potential and are unlikely to progress to malignancy if stable and devoid of other concerning features. Among all scans performed, 0.08% of patients had a cyst larger than 3 cm—a worrisome feature per the IAP guidelines—or larger than 4 cm, which is a relative surgical indication under European guidelines.^8^ In our sample of individuals undergoing whole-body screening MRI, 7.0% of individuals were found to have an incidental PCL, with only 17 cysts measuring above these size thresholds. While most of the cysts were small and unlikely to require intervention, their presence reinforces the high prevalence of radiographically visible preclinical findings in asymptomatic individuals. Notably, the size criteria are based on data with a low false-negative rate but a high false-positive rate, suggesting that many patients may undergo long-term surveillance or interventions without clear benefit. Meanwhile, approximately 1500 (7%) of individuals with smaller cysts will have initiated years of additional testing and follow-up, despite most of these lesions having little to no clinical importance.

### Limitations

This study has several important limitations that warrant consideration. First, the cohort consisted of individuals who voluntarily elected to undergo commercial whole-body MRI screening, introducing the potential for selection bias. This population likely represents a self-selected group with heightened health awareness, access to discretionary health care spending, or concern about underlying disease, and therefore may not be fully representative of the general population. As a result, the external validity of these findings may be limited. While this self-selected nature may introduce selection bias or limit generalizability, this concern has been in part mitigated by performing age and sex standardization using US Census population weights. Further, by leveraging a large cohort of more than 21 000 individuals, this study helps mitigate some of the selection biases inherent to screening-based analyses and provides more stable prevalence estimates than prior screening MRI studies conducted in smaller, relatively homogeneous populations.^19,20,21,22^

Second, a whole-body MRI screening protocol was used in this study. This may have implications for the detection of very small PCLs compared with pancreatic-specific sequences, as was alluded to in a study using 3T MRI plus magnetic resonance cholangiopancreatography (MRCP).^21^ However, a prior study^10^ also demonstrated that whole-body MRI has good sensitivity for detecting clinically relevant pancreatic cysts and that the prevalence of PCLs with or without MRCP sequences is not significantly different. Thus, it is possible that pancreatic protocol MRI may identify a higher prevalence of PCLs. Similarly, cases in which no cysts were identified in the reports were not reviewed a second time; thus, there is a possibility of very small lesions being underreported. In both these cases, prevalence could be higher than what is reported.

Third, the cross-sectional nature of the study provides a snapshot in time and precludes assessment of cyst evolution, growth, or malignant potential over time. Importantly, cyst size was used in this study as a pragmatic and reproducible surrogate for risk assessment within a screening setting, since whole-body MRI examinations are often not optimized for detailed assessment of other high-risk features (ie, mural nodularity, solid components, cyst subtype), especially in very small PCLs, which constitute most lesions that were incidentally identified. Thus, longitudinal imaging and identification of other worrisome or high-risk features would be required to truly determine the natural history of incidentally detected cysts in this population. Acknowledging these limitations, this study provides valuable insight into the prevalence of PCLs and clinically relevant size measurements at time of presentation in a large screening cohort.

Fourth, while the advent of whole-body MRI as a tool for preventive health screening has demonstrated utility in revealing early-stage malignant neoplasms in asymptomatic individuals,^34,35^ increased cross-sectional imaging may also lead to the discovery of incidentalomas. Due to the known malignant potential of PCLs, their inadvertent identification generates anxiety for patients and raises questions regarding subsequent imaging, invasive testing, and surgery.^36^ While guidelines from several governing bodies have directed the care algorithms for PCLs, the current processes require significant resources and carry the potential to overtreat low-grade cysts.^37,38^ For example, in a 2018 study of major pancreatectomy for IPMN in the postguideline era,^38^ almost 80% of tumors were found to harbor only low-grade dysplasia on final pathological evaluation.

Thus, it is critical to risk stratify PCLs to highlight lesions that require additional characterization while minimizing the downstream health care burden associated with serial diagnostics and/or resections.^17^ Our results highlight the need for data-driven approaches to stratify risk and personalize follow-up, ensuring that screening efforts remain both clinically effective and cost-efficient. For high-risk lesions, further assessment with endoscopic ultrasonography and fine needle aspiration may be warranted,^13^ as well as cyst fluid analyses of molecular markers for diagnosis and risk stratification,^39,40,41^ which can be used alongside clinical characteristics of lesions. As screening technologies advance, the future of preventive imaging will depend not only on the ability to detect subclinical or indolent lesions, but also on the capacity to implement proportionate, evidence-based management strategies that balance early detection without causing unnecessary surveillance or intervention. As the general population continues to undergo preventive screening, PCLs will be increasingly discovered.

## Conclusions

In this cross-sectional study of 21 651 individuals undergoing whole-body screening MRI, 7.0% of individuals had an incidental PCL, with very few 3 cm or larger. Older age, female sex, family history of PDAC, alcohol use, and Latin American or Middle Eastern ethnicity were associated with an increased likelihood of cyst development. Novel strategies for risk stratification of PCLs are warranted.

## Notes

Supplement.
Data Sharing Statement
