# Supplementary material for: Prevalence and Size-Based Risk Categorization of Pancreatic Cysts Among Asymptomatic Individuals With Screening MRI
Source: JAMA Netw Open. 2026 Mar 9;9(3):e260983. doi: 10.1001/jamanetworkopen.2026.0983 (PMC12973103; doi:10.1001/jamanetworkopen.2026.0983)
Supplement: Supplement. — Data Sharing Statement [file jamanetwopen-e260983-s001.pdf]

Data Sharing Statement

Wong. Prevalence and Size-Based Risk Categorization of Pancreatic Cysts Among Asymptomatic Individuals With Screening MRI. *JAMA Netw Open*. Published March 09, 2026. doi:10.1001/jamanetworkopen.2026.0983

Data
Data available: Yes
Data types: Data dictionary
How to access data: paul.wong@ucsf.edu
When available: With publication

Supporting Documents
Document types: None

Additional Information
Who can access the data: researchers whose proposed use of the data has been approved
Types of analyses: statistical analysis
Mechanisms of data availability: with a signed data access agreement with us and Prenuvo
